# Biological Activities and Proteomic Profile of the Venom of *Vipera ursinii* ssp., a very Rare Karst Viper from Croatia

**DOI:** 10.3390/toxins12030187

**Published:** 2020-03-16

**Authors:** Maja Lang Balija, Adrijana Leonardi, Marija Brgles, Dora Sviben, Tihana Kurtović, Beata Halassy, Igor Križaj

**Affiliations:** 1 University of Zagreb, Centre for Research and Knowledge Transfer in Biotechnology, Rockefellerova 10, HR-10 000 Zagreb, Croatia; maja.langbalija@gmail.com (M.L.B.); mbrgles@gmail.com (M.B.); dora.sviben@gmail.com (D.S.); tihana.kurtovic@unizg.hr (T.K.); 2 Jožef Stefan Institute, Department of Molecular and Biomedical Sciences, Jamova cesta 39, SI-1000 Ljubljana, Slovenia; adrijana.leonardi@ijs.si

**Keywords:** snake venom, *Vipera ursinii* ssp., karst viper, toxicity, venomics, insecticide

## Abstract

The karst viper (*Vipera ursinii* ssp.) favours high-mountain dry grasslands in southern and south-eastern Croatia. It is medically less important than other *Vipera* species, because of its remote habitat and the very small amount of venom that it injects by its relatively short fangs. The scientific literature on *Vipera ursinii* deals mostly with the morphology, ecology and distribution range of this snake, due to the species’ conservation issues, while the toxinological aspects of its venom have not so far been investigated. Here we report on the composition and biological activity of the *Vipera ursinii* ssp. venom. Using a proteomics approach, we have identified 25 proteins in the venom that belong to seven protein families: snake venom metalloproteinase, serine protease, secreted phospholipase A_2_, cysteine-rich secretory protein, snake C-type lectin-like protein, serine protease inhibitor and nerve growth factor. The *Vipera ursinii* ssp. venom was found to be distinctively insecticidal. Its lethal toxicity towards crickets was more than five times greater than that of *Vipera ammodytes ammodytes* venom, while the opposite held in mice. Interestingly, the mode of dying after injecting a mouse with *Vipera ursinii* ssp. venom may suggest the presence of a neurotoxic component. Neurotoxic effects of European vipers have so far been ascribed exclusively to ammodytoxins and ammodytoxin-like basic secreted phospholipases A_2_. Structural and immunological analyses of the *Vipera ursinii* ssp. venom, however, confirmed that ammodytoxin-like proteins are not present in this venom.

## 1. Introduction

Orsini’s viper, *Vipera ursinii*, is one of the most endangered snake species in Europe. Currently, four subspecies are officially recognized [1], but an additional one may be introduced based on a recent mitochondrial DNA analysis [2]. Three of them, *V. u. ursinii*, *V. ursinii* ssp. and *V. u. macrops*, prefer living at higher altitudes in mountain grasslands (karst vipers), while *V. u. rakonsiensis* and *V. u. moldavica* prefer lowlands (meadow vipers). The distribution area of *V. ursinii* is highly fragmented and covers south-western France, central Italy, Hungary, Romania as well as western and eastern parts of the Dinaric Mountains. Due to the threat of extinction, the Council of Europe adopted, in 2005, the *V. ursinii* protection plan [3].

**Figure 1:**
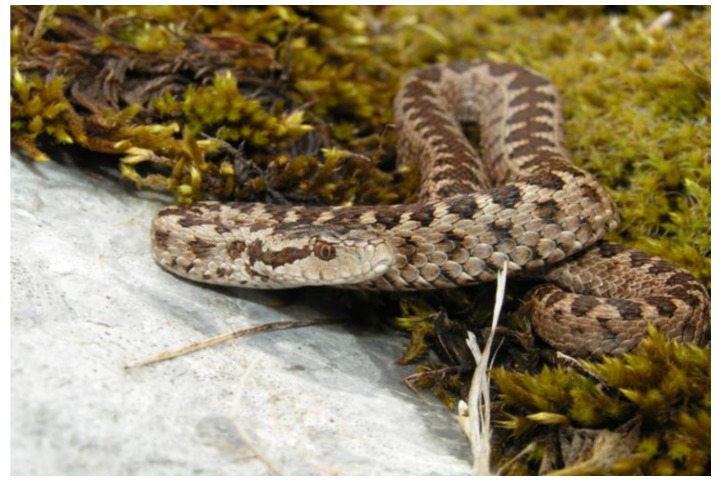
The karst viper (*V. ursinii* ssp.) is a short, light grey or brownish snake characterized by a dark zigzag pattern along its back. This one was photographed in southern Velebit, Croatia (photo by Dušan Jelić, and we got the copyright permission of Dušan Jelić).

*V. ursinii* ssp. (Figure 1) is a poorly described, novel subspecies that lives in the north-western part of the Dinarides (Croatia, Bosnia and Herzegovina). It is genetically separated from the other karst vipers that inhabit the Dinaric Mountains all the way to Albania, and is more like the West European subspecies [1,4]. The Croatian *V. ursinii* ssp. is a highly endangered viper that is found in five isolated localities: in southern Velebit, Poštak, Dinara, Troglav and the Kamešnica mountains, at altitudes above 1100 m [5,6]. It is the smallest venomous snake in Croatia, reaching an average size of 54 cm. Typically, its head is clearly separated from its neck and the body is short and smooth. The grey or brownish ground colour is usually lighter dorsally. In most cases, the animal is also characterized by a dark zigzag pattern along its back. The snake is entomophagous, i.e., its typical diet comprises insects (grasshoppers and crickets) [7]. People rarely encounter this snake, so accidents are very scarce. In addition, the snake has very short fangs (2–3 mm) through which it can inject only minute amounts of venom (1–4 mg). Accordingly, the symptoms reported in patients envenomed by the *V. ursinii* venom have been very mild and local, and were resolved spontaneously without medical intervention [8]. 

The scientific literature on *V. ursinii* deals mostly with its morphology, ecology and distribution, addressing, in the first place, its preservation problems [1,3,9,10], while its venom composition and toxic activities have been only poorly investigated. Regarding the latter, the venom of *V. ursinii* was reported, in mice, to be haemorrhagic but not myotoxic [11]. The mRNA transcripts of two secreted phospholipases A_2_ (sPLA_2_s), ammodytin I_1_ (AtnI_1_) and ammodytin I_2_ (AtnI_2_), were identified in the *V. ursinii* venom glands [12]. 

To obtain an insight into the functioning at the molecular level of the venom of this rare and ecologically very distinctive snake, we report here a toxinological description of the venom along with its comprehensive proteomic analysis. This opened the way to unravelling a unique insecticidal activity, leading potentially to new pesticides. 

## 2. Results

### 2.1. Toxinological Characterisation of V. ursinii ssp. Venom

The lethal toxicity of *V. ursinii* ssp. venom was determined in mice and crickets. *V. ursinii* ssp. venom was less toxic than *V. a. ammodytes* venom in mice—its average LD_50_ was more than 4 times higher than that of *V. a. ammodytes* venom (Table 1). Clinical pictures of mice dying from *V. ursinii* ssp. or *V. a. ammodytes* venom were similar. After i.v. application of venom, animals rapidly showed signs of anxiety and severe dyspnoea, followed by convulsions and ataxia with spasms. Finally, they died. Such symptoms are characteristic of neurotoxins or stroke. The two venoms, however, differ distinctly in that *V. ursinii* ssp. venom induced sudden and sporadic, dose-unrelated deaths when injected in experimental animals, while *V. a. ammodytes* venom did not.

Both venoms were also lethally toxic for crickets. In contrast to the results in mice, *V. ursinii* ssp. venom displayed higher toxicity in crickets than *V. a. ammodytes* venom. The average LD_50_ of the first was more than 5-fold lower than that of the latter (Table 1). Interestingly, for both types of animals, the LD_50_ values are in the same µg range. The species selectivity of a particular venom is clear –comparing mass-normalized LD_50_s, *V. a. ammodytes* venom was almost 114-fold more lethal for mice than for crickets, while *V. ursinii* ssp. venom was only 5-fold (Table 1).

The haemorrhagic activities of the two venoms in rats were similar (Table 1).

### 2.2. Immunological Cross-Reactivity between V. ursinii ssp. and V. a. ammodytes Venoms

**Figure 2:**
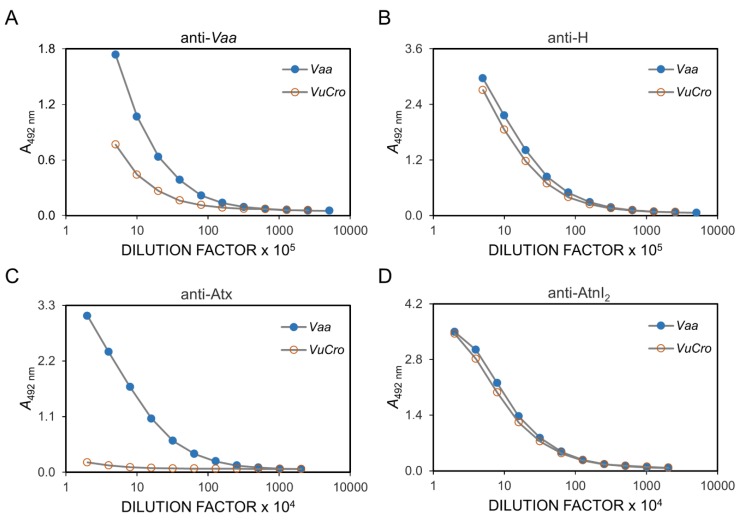
Antigen similarity of *V. ursinii* ssp. and *V. a. ammodytes* venoms by ELISA. The venoms of the Croatian *V. ursinii* ssp. (*Vu*Cro) and *V. a. ammodytes* (*Vaa*) were compared by ELISA assay using rabbit antibodies raised against the whole *V. a. ammodytes* venom (anti-*Vaa*) (**A**), the pool of *V. a. ammodytes* haemorrhagins (anti-H) (**B**), *V. a. ammodytes* neurotoxic sPLA_2_ Atx (anti-Atx) (**C**) and *V. a. ammodytes* non-toxic sPLA_2_ AtnI_2_ (anti-AtnI_2_) (**D**). All sera, except anti-Atx, recognized components of the *V. ursinii* ssp. venom, suggesting that neurotoxic Atx is absent in this venom. Antibody binding was detected by incubation with HRP-anti-rabbit IgG and the subsequent OPD-based colour reaction.

**Figure 3:**
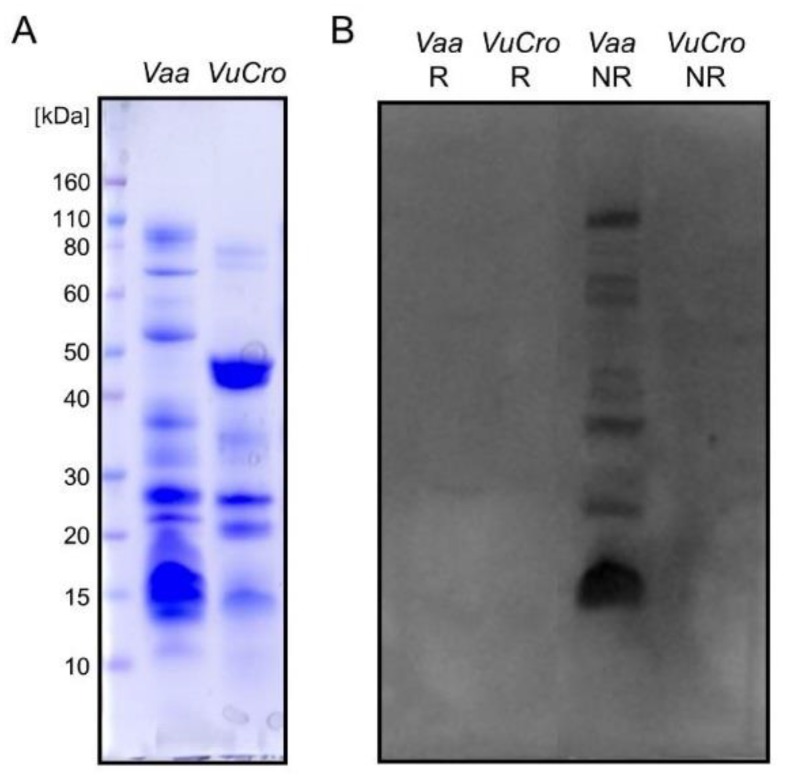
WB analysis of *V. ursinii* ssp. and *V. a. ammodytes* venoms using anti-Atx serum. Crude venoms of the Croatian *V. ursinii* ssp. (*Vu*Cro) and *V. a. ammodytes* (*Vaa*) (40 µg/lane) were analysed on 4–12% SDS-PAGE gel under non-reducing (**A**) and reducing conditions. Proteins in gels were either stained with CBB R250 (**A**) or electro-transferred in tank onto the PVDF membrane and immuno-stained using anti-Atx serum and an ECL detection system (**B**). On Western blots, signals appeared only in the case of the *V. a. ammodytes* venom sample under non-reducing conditions, thus confirming the absence of Atx-like proteins in the *V. ursinii* ssp. venom.

Rabbit antisera against the whole *V. a. ammodytes* venom (anti-*Vaa*) and its various components, the pool of *V. a. ammodytes* haemorrhagins (anti-H), *V. a. ammodytes* neurotoxic sPLA_2_ Atx (anti-Atx) and *V. a. ammodytes* non-toxic sPLA_2_ AtnI_2_ (anti-AtnI_2_) were used to probe cross-reactivity between the *V. ursinii* ssp. venom components, using WB and ELISA assays. Anti-*Vaa*, anti-H and anti-AtnI_2_ antibodies proved to be paraspecific in ELISA tests. On the other hand, anti-Atx antibodies did not recognize the *V. ursinii* ssp. venom-coated ELISA plate at all (Figure 2). ELISA results were confirmed by WB analysis. As shown in Figure 3, Atx-like sPLA_2_s were not detected using Atx-specific antibodies in *V. ursinii* ssp. venom.

### 2.3. Proteomics of V. ursinii ssp. Venom and Its Comparison with V. a. ammodytes Venom

#### 2.3.1. Proteome of *V. ursinii* ssp. Venom

**Figure 4:**
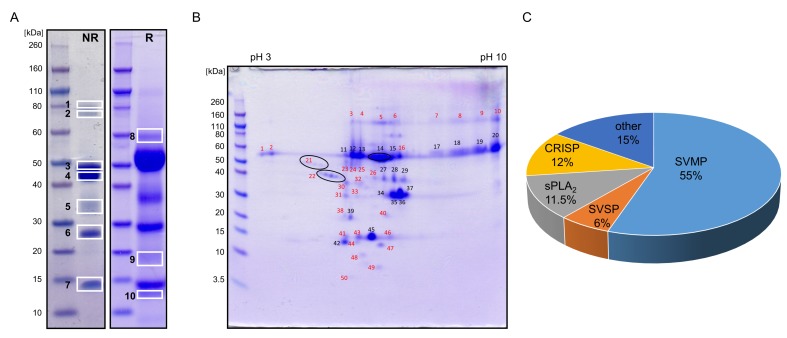
Profiling of the protein composition of *V. ursinii* ssp. venom of snakes living in the wild. (**A**) The *V. ursinii* ssp. venom (40 µg/well) was analysed by SDS-PAGE under non-reducing (NR) and reducing (R) conditions on 4–12% Bis-Tris gel. (**B**) 250 µg of the venom was separated in two dimensions, using a 7 cm IPG strip, pH 3–10, in the first, and 4–12% Bis-Tris gel in the second dimension. Proteins in the gels were stained with CBB R250. The indicated bands (white frames) and spots (red numbers) were excised and trypsinized. The obtained peptides were extracted from the gel and analysed by ESI–MS/MS. Black numbers on the 2-DE gel denote proteins that were identified by ESI–MS/MS of 1-DE bands (**A**). (**C**) Relative abundance (% of the total venom protein content) of major protein families in *V. ursinii* ssp. venom was estimated from intensities of the 2-DE spots in which they were detected. SVMP, snake venom metalloproteinase; CRISP, Cys-rich secretory protein; sPLA_2_, secreted phospholipase A_2_; SVSP, snake venom serine protease.

To analyse the *V. ursinii* ssp. venom proteome, the crude venom collected from the wild snakes was separated by 1-DE in the molecular mass range 10 to 80 kDa (Figure 4A), and by 2-DE in the isoelectric point (pI) range of 3 to 10 and a molecular mass range of 3.5 to 110 kDa (Figure 4B). *V. ursinii* ssp. venom was resolved into seven and eight distinct bands on non-reducing and reducing 1-DE, respectively. 

More than 50 protein spots were observed on the 2-DE gel. We assumed that proteins from the most intense bands on the 1-DE gel were those appearing as the major spots in the 2-DE gel at the same molecular masses, e.g., at ~50, ~35, ~30 and ~15 kDa. Proteins from the 1-DE Bands 3 and 4 thus constituted mostly 2-DE Spots 11–20. The major protein from Band 5 was located in 2-DE Spots 27 to 29, the one from Band 6 in 2-DE Spots 34 to 37, and proteins from Band 7 mostly in 2-DE Spots 42 and 45. For the MS analysis, dominant protein bands were thus excised from the non-reduced 1-DE gel (7 bands labelled on the gel in Figure 4A). Less abundant protein bands were excised from the reduced 1-DE gel (3 bands labelled 8 to 10 on the gel in Figure 4A) and picked from the 2-DE gel (31 spots designated by red numbers in Figure 4B). 

Again, we assumed that the main protein of 1-DE Band 9 at ~18 kDa is the same as the protein in 2-DE Spot 39, the most intense spot at the same molecular mass. The proteins were trypsinized in-gel and the resulting peptides extracted and analysed by LC–ESI–MS/MS. Altogether, in selected bands and spots, 25 proteins were identified. They can be grouped into seven protein families: snake venom metalloproteinase (SVMP), snake venom serine protease (SVSP), sPLA_2_, cysteine-rich secretory protein (CRISP), snake C-type lectin-like protein (snaclec), Kunitz-type serine protease inhibitor (KSPI) and venom nerve growth factor (VNGF) family (Table S1). No proteins could be identified in Spots 4, 6, 8, 9, 40, 49 and 50, due either to too low amounts of material or to the lack of similar/identical sequences in the protein database used to search the MS spectra. The most abundant were proteins of ~50 kDa belonging to the SVMPs that constituted 1-DE Bands 1 to 4 and 8 (corresponding to 2-DE Spots 11 to 20), and 2-DE Spots 1 to 5, 10 and 23 to 25. The second most abundant were CRISPs, which were identified as the major components of 1-DE Band 6 with apparent molecular masses of ~27 kDa (corresponding to 2-DE Spots 34 to 37). CRISP was also detected as a minor constituent of the 1-DE bands at higher apparent molecular masses of 43 kDa (Band 4) and 35 kDa (Band 5), as well as 50 kDa (2-DE Spot 16). sPLA_2_s were identified in the 1-DE Band 7 and 2-DE Spots 41 to 47, while SVSPs were found mostly in 1-DE Band 5 (corresponding to 2-DE Spots 27 to 29), and in 2-DE Spots 21, 22 and 30. Two KSPIs were identified in 1-DE Band 10, one as a homologue of chymotrypsin inhibitor (0909196A) from *V. a. ammodytes* and the other as a trypsin inhibitor Vur-KIn (P0DKL8) from the closely related snake *V. renardi*. Relative abundances (expressed in % of the total venom protein) of major protein families in the *V. ursinii* ssp. venom were estimated from the 2-DE spot intensities. As evident from Figure 4C, SVMPs are the most abundant proteins in the *V. ursinii* ssp. venom, followed by CRISPs, sPLA_2_s and SVSPs. 

**Figure 5:**
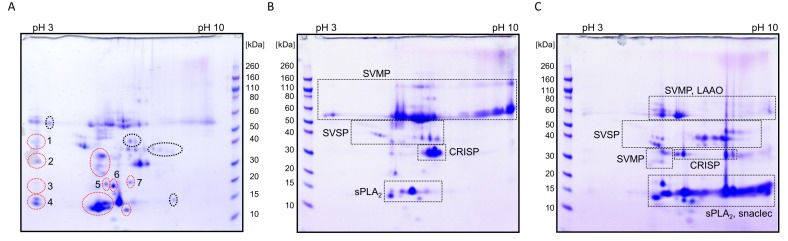
Comparative 2-DE analyses of *V. ursinii* ssp. and *V. a. ammodytes* venoms. The 2-DE profile of the Croatian *V. ursinii* ssp. venom collected from wild snakes (*Vu*Cro) (**B**) was compared with that of captured *V. ursinii* ssp. snakes (*Vu*Cro-c) (**A**) or that of the *V. a. ammodytes* venom (**C**). Distribution of the dominant protein families in the *V. ursinii* ssp. and in the *V. a. ammodytes* venom is framed. Additional (Spots 1–7) or more abundant protein spots in the *Vu*Cro-c venom relative to the *Vu*Cro venom are encircled red. Proteins identified in Spots 1–7 (**A**) are presented in Table S2. In spots encircled black no proteins were identified.

For conservation and research purposes, some *V. ursinii* ssp. specimens are kept in captivity, where they experience a food regime different from that in the wild. Namely, captive animals were fed on mice, while, in their natural habitat, they mostly eat insects. In order to examine how different diets influence the venom composition, venoms collected from the snakes in captivity (*Vu*Cro-c) and those obtained from wild animals (*Vu*Cro) were analysed comparatively by 2-DE (Figure 5A,B). Differences were observed mainly in the acidic part of the 2-DE gels where, in the case of the *Vu*Cro-c venom, certain spots appeared more intense than those in *Vu*Cro venom. In addition, in this area, several additional spots are present in the case of *Vu*Cro-c venom (red encircled Spots 1 to 7 in Figure 5A). Proteins identified in these spots are listed in Table S2. Spots of the increased intensity in *Vu*Cro-c regarding to the *Vu*Cro venom (Figure 5A,B) correspond to the Spots 30, 38, 39, 40 and 42 in Figure 4B. In these spots, proteins belonging to SVSPs, sPLA_2_s, SVMPs, snaclecs, VNGFs and KSPIs were identified (Table S1). Of these, sPLA_2_s appeared much more highly abundant in the venom of animals in captivity (*Vu*Cro-c) than in the venom of those living in the wild (*Vu*Cro). In Table 2 the proteins are listed whose abundance is distinctively different in *Vu*Cro-c and *Vu*Cro venoms.

#### 2.3.2. Comparative Analysis of *V. ursinii* ssp. and *V. a. ammodytes* Venoms

Comparative 2-DE analysis of the crude *V. ursinii* ssp. and *V. a. ammodytes* venoms exposed large differences in their composition with respect to distribution and intensity of most of the protein spots (Figure 5B,C). The *V. ursinii* ssp. proteome was compared with the recently published proteome of the *V. a. ammodytes* venom [15]. In general, there are far fewer basic proteins in the *V. ursinii* ssp. venom than in the *V. a. ammodytes* venom. Further, in the *V. ursinii* ssp. venom, high molecular mass proteins prevail over those with low molecular masses and vice versa in the case of the *V. a. ammodytes* venom. Thus, high molecular mass SVMPs are the major constituents of the *V. ursinii* ssp. venom, while low molecular mass sPLA_2_s and snaclecs dominate in the *V. a. ammodytes* venom. The latter are scarce in the *V. ursinii* ssp. venom, which is also completely devoid of basic sPLA_2_s. SVSPs are found in many spots in both venoms, but in much higher quantities in the *V. a. ammodytes* venom, where they are also much more diverse regarding their pIs. In contrast, the CRISPs are more abundant in the *V. ursinii* ssp. than in the *V. a. ammodytes* venom. LAAOs were not detected in the *V. ursinii* ssp. venom. LAAOs are usually the components that colour snake venoms yellow. In accordance with our results, the colour of *V. ursinii* ssp. venom is only slightly yellow.

## 3. Discussion

*V. ursinii* ssp. is one of the rarest snakes in Europe. Endangered by extinction, it can be found only in certain mountain regions in Croatia and Bosnia and Herzegovina, at altitudes exceeding 1000 m. Due to the difficulty in encountering this snake in the wild, its venom remained practically unstudied until this work.

Due to its extreme habitat, *V. ursinii* ssp. has a special diet consisting mostly of insects. The adaptation of the snake venom to this prevailing food is clear. Compared to *V. a. ammodytes*, which feeds predominantly on rodents, the venom of *V. ursinii ssp.* displayed a more than 5-fold higher lethal toxicity in crickets but more than 4-fold lower toxicity in mice (Table 1). Such an adaptation of venom efficacy to diet has been reported before. For example, the venoms of vipers that are not entomophagous (*V. berus* and *V. nikoloski*) have a lower toxicity for crickets than those of pronouncedly entomophagous vipers (*V. renardi* and *V. lotievi*) [7]. In addition, the average mass-normalized LD_50_ values that we determined in crickets for *V. ursinii* ssp. and *V. a. ammodytes* venoms (Table 1) were comparable with those reported in [7] for entomophagous and non-entomophagous snakes, ~10 and ~30 µg/g, respectively.

The lethal toxicities of both *V. ursinii* ssp. and *V. a. ammodytes* venoms were higher in mice than in crickets (Table 1). Higher lethality for mice than for arthropods has also been observed in the case of other viper venoms, e.g., those of the Pelias group of snakes [7] and *Echis* spp. [16,17]. This points to the fact that, during evolution, rodents have been the principal food of vipers, with other species like insects or scorpions being secondary. Snakes of other families may have developed other food preferences and, correspondingly, venoms with other prey specificities. For example, the venom of a colubrid, the brown tree snake (*Boiga irregularis*), is more toxic to birds and lizards than to mammals [18]. However, the mass-adjusted LD_50_ of the *V. a. ammodytes* venom was more than 100-fold lower in mice than in crickets, while that of the *V. ursinii* ssp. venom was just 5-fold lower. This shows that *V. ursinii* ssp. adapted to their new living environment in which insects prevail over mice as the available food. The snake, therefore, has been forced to turn from being a mouse-hunting to an insect-hunting animal. 

Gel-based proteomic analysis of the venom of the wild *V. ursinii* ssp. species revealed a proteome much simpler than that of the recently published *V. a. ammodytes* venom proteome [15]. Almost two times fewer proteins were identified in the *V. ursinii* ssp. than in the *V. a. ammodytes* venom. While the proteins in the venom of *V. ursinii* ssp. represent seven different protein families, representatives of 16 protein families were found in the venom of *V. a. ammodytes*. Both venoms contain SVMPs, SVSPs, sPLA_2_s, CRISPs, snaclecs, VNGFs and KSPIs, which are typical of most viperid snake venoms [19,20]. In general, the number of homologues, representatives of the most abundant enzyme families, SVMPs, SVSPs and PLA_2_s, is much lower in the *V. ursinii* ssp. than in the *V. a. ammodytes* venom. Furthermore, snaclecs appear only as covalently bound SVMP subunits in the *V. ursinii* ssp. venom. A similar observation was reported in the venomic study of *V. b. berus* [21]. In the *V. a. ammodytes* venom, however, snaclecs are also present as an abundant and structurally diversified group of disulphide-linked heterodimeric proteins. Members of the two protein families that are usually present in viperid venoms, LAAOs and disintegrins, were not identified in the *V. ursinii* ssp. venom. So far, of the genus Vipera, only *V. anatolica*, has been reported to be devoid of LAAOs and *V. nikolskii* of disintegrins [20]. 

The dominant components of *V. ursinii* ssp. venom are SVMPs, representing 55% of the total venom proteins (Figure 4C). Among snakes of subfamily Viperinae, to which *V. ursinii* ssp. belongs, a similar prevalence of SVMPs was also observed in venoms of genera *Cerastes* and *Echis*, and *M. lebetina* from Tunisia [20]. SVMPs are structurally and functionally highly diverse zinc-dependent enzymes [22,23]. The *V. ursinii* ssp. venom displays high haemorrhagic activity. Its MHD in rat is similar to that displayed by the *V. a. ammodytes* venom (Table 1). The predominance of the P-III class SVMPs in the *V. ursinii* ssp. venom explains its haemorrhagic nature. The high haemorrhagic potential of the P-III SVMPs is based on the presence of non-catalytic domains (disintegrin-like and Cys-rich) in these molecules that mediate their binding to specific substrates in the microvasculature [24]. Haemorrhagic SVMPs namely cause blood microvessel damage by hydrolysing the key proteins in the basement membrane of the capillary wall particularly type IV collagen. This leads to weakening and disruption of the wall due to the hemodynamic pressure. In accordance with the high degree of immunological cross-reactivity of SVMPs from *V. ursinii* ssp. and *V. a. ammodytes* venoms (Figure 2), we have identified *V. ursinii* ssp. haemorrhagins, mostly as homologues of those described in the *V. a. ammodytes* venom, VaH3, Vaa-MPIII-1, Vaa-MPIII-3 and Vaa-MPIII-4 (Table S1). Of these, VaH3 has been characterized as one of the main haemorrhagic factors of the *V. a. ammodytes* venom [25]. As an N-glycosylated homodimer, it belongs to the P-IIIc subclass of SVMPs. In vitro, VaH3 also markedly prolonged blood coagulation, due to its fibrinogenolytic activity, but only weakly affected platelet aggregation. Vaa-MPIII-1 and Vaa-MPIII-4 are present in trace amounts in the *V. a. ammodytes* venom [15]. In contrast, in the *V. ursinii* ssp. venom, these two SVMPs have been identified in several 1-DE bands and 2-DE spots (Figure 4, Table S1). Moreover, Vaa-MPIII-1 appears to be the major SVMP of the *V. ursinii* ssp. venom. Comparison of the Vaa-MPIII-1 and Vaa-MPIII-4 primary structures with those of the other SVMPs whose activities are known, revealed their highest identities (~75%) with the haemorrhagic SVMPs from viperid venoms. This suggests that Vaa-MPIII-1 and Vaa-MPIII-4 also possess haemorrhagic activity. Vaa-MPIII-3 is a member of the recently introduced P-IIIe SVMP subclass. That subclass evolved from the classical P-III SVMPs by loss of the MP domain [15], thus consisting of only a complete or a partial disintegrin-like domain and a Cys-rich domain. Interestingly, the *V. ursinii* ssp. Vaa-MPIII-3 peptides were found in the high molecular mass protein spots (48 to 120 kDa), suggesting the existence of a Vaa-MPIII-3-like molecule also containing the MP domain. 

The pathologic effect of haemorrhagins can be exacerbated by the action of other venom components that interfere with the processes of blood coagulation and/or platelet aggregation [22]. For example, the extensive and uncontrolled activation or degradation of coagulation factors by SVMPs and SVSPs leads to a consumptive coagulopathy [26]. Potentially having such a role, we found, in the *V. ursinii* ssp. venom, homologues of two SVMP activators of the coagulation factor X (FX), VLFXA from *M. lebetina* (AAQ17467) and RVV-X from *D. russelii*. The corresponding proteins in the *V. a. ammodytes* venom are VAFXAs [27]. All the aforementioned FX activators belong to the P-IIId subclass, in which a dimeric snaclec (light chain) is disulphide-linked to the Cys-rich domain of a P-III SVMP (heavy chain). The snaclec subunit serves to bind the protease to the Gla-domain of FX [28], thus positioning it correctly for effective proteolytic activation of the factor. 

We have also identified several members of the SVSP family in the *V. ursinii* ssp. venom that may have pro- and anti-coagulant activities. Those being potentially procoagulant are *V. ursinii* ssp. homologues of RVV-V from the *Daboia siamensis* venom [29] and Vaa-SP-VX from the *V. a. ammodytes* venom (Latinović et al., unpublished), which activate FV. In contrast to the SVMP activators of blood coagulation factors, the SVSP activators are usually single-chain molecules with three-dimensional structures that resemble the structure of physiological activators, e.g., the heavy chain of the activated blood coagulation factor X (FXa) and thrombin, though with sequence replacements that confer high substrate specificity and resistance towards the natural inhibitors, the serpins [30]. The most abundant *V. ursinii* ssp. SVSP is a homologue of an anticoagulant *V. a. ammodytes* protein, Vaa-SPH-1 (KT148824). The latter is an enzymatically inactive SVSP that inhibits the activity of the intrinsic tenase complex by binding to FVIIIa in place of FIXa [31]. 

Like SVSPs, venom sPLA_2_s can also affect haemostasis in a catalytic activity-independent and/or dependent way [22]. Ammodytoxins (Atx), basic sPLA_2_s from the *V. a. ammodytes* venom, inhibit blood coagulation by binding to FXa, thereby preventing formation of the prothrombinase complex. They have not been detected in the *V. ursinii* ssp. venom. Ammodytins (Atn), acidic sPLA_2_s from the *V. a. ammodytes* venom, induce anticoagulant and antiplatelet effects by hydrolysing phospholipids [32]. AtnI_1_ and AtnI_2_ are present in the *V. ursinii* ssp. venom, AtnI_1_ (CAE47156) being the major isoform. AtnI_1_ proteins from the *V. ursinii* ssp. and *V. a. aspis* (AAN59986) venoms are practically identical (99% sequence identity). The latter was characterized as being indirectly haemolytic and weakly anticoagulant [33], and the same activities can be expected in the case of the *V. ursinii* ssp. enzyme. On the other hand, *V. ursinii* ssp. AtnI_2_ is probably endowed with a potent haemostatic activity based on 96% sequence identity with Vur-PL2 (ADG86231) from the closely related Russian snake *V. renardi* (former *V. ursinii renardi*). Vur-PL2 was namely described as a strongly anticoagulant and platelet aggregation inhibitory enzyme [34]. Although only weakly toxic in mice (LD_50_ = 10.7 mg/kg), one third of experimental animals died from internal bleeding in various organs at an LD_50_ dose of Vur-PL2.

Although the *V. ursinii* ssp. venom was much less toxic to mice than the *V. a. ammodytes* venom, the mode of dying of animals after they received a lethal dose of one or other of the venoms was frequently similar, suggesting neurotoxicity as the cause of death. Unusually, the neurotoxic-like effects did not take place in every animal envenomed by the *V. ursinii* ssp. venom, and, when it did take place, they were apparently independent of the received dose of the venom. Such phenomena have never been observed when poisoning mice with the *V. a. ammodytes* venom, which suggests different molecular backgrounds of the effects induced by the two venoms. In the case of *V. a. ammodytes* and related European viper venoms, the neurotoxicity stems from basic sPLA_2_s such as Atx [35]. The latter were not found in the *V. ursinii* ssp. venom when using alternative analytical approaches (Figure 2, Figure 3A and Figure 5). A similar situation, i.e., sporadic induction of symptoms considered as neuropathological (e.g., paraesthesia) in spite of the absence of basic sPLA_2_s in the venom, was also reported for envenomation by venom from *V. u. ursinii* in south-eastern France [12]. If the effects were indeed neurotoxic then they cannot be induced by neurotoxic sPLA_2_s. Sudden and sporadic, dose-unrelated deaths of animals injected with *V. ursinii* ssp. venom, however, offered also another possible explanation—a stroke, induced by coagulopathic venom components following the i.v. application of the venom. 

The non-enzymatic proteins found in the *V. ursinii* ssp. venom are members of the CRISP, VNGF and KSPI protein families, all non-lethal constituents of snake venoms. The CRISP family is represented by homologues of Vaa-CRISP-1 (KT148819) and Dr-CRPK from the *D. russelii* venom (ACE73567). These two proteins share 90% sequence identity but neither has a known biological function. Although CRISPs are widespread in snake venoms, knowledge concerning their biological targets and activities is limited. Many so-far characterised CRISPs either modulate the conductivity of various ion channels [36] and induce inflammation [37,38] or have antiprotozoal [39] or antiangiogenic [40] activity. 

VNGFs are common components of snake venoms [41] and the *V. ursinii* ssp. venom is not an exception. These molecules are either covalent or non-covalent homodimers expressing nerve growth-promoting activity. Some of them, like the non-covalent homodimer from *M. lebetina* venom (P25428), are glycoproteins. *V. ursini*’s VNGF (AEH59582) shares 97% sequence identity with the *M. lebetina* VNGF and has a conserved N-glycosylation site at Asn148. The apparent monomer mass of both *V. ursinii* ssp. and *M. lebetina* VNGF proteins is ~18 kDa (Figure 3A, Table S1). This suggests that *V. ursinii* ssp. VNGF is also glycosylated.

Venoms of Viperidae and Elapidae snakes contain ~60 amino acid-long Kunitz-type SPIs (KSPIs). We have also identified such molecules in the *V. ursinii* ssp. venom as homologs of VaaChi, a KSPI from the *V. a. ammodytes* venom [42], and Vur-KIn, a KSPI from the *V. renardi* venom [12]. The structural fold of KSPIs resembles that of the bovine pancreatic trypsin inhibitor [43]. Three KSPIs from the *V. a. ammodytes* venom have already been characterised, two as potent inhibitors of trypsin (VaaTi) and as the inhibitor of chymotrypsin (VaaChi) [42]. VaaTi also inhibited plasmin and plasma kallikrein, while VaaChi was shown to form a non-covalent complex with Atx, increasing its toxicity [44]. The activity of Vur-KIn has not yet been determined but it shows the highest sequence identity (≥80%) with trypsin inhibitors from Viperinae snakes, VaaTi from *V. a. ammodytes* (P00991), PPTI from *Pseudocerastes persicus* (C0HLB2) and a KSPI from *Eristicophis macmahoni* (P24541). Further supporting the trypsin inhibitory activity of Vur-KIn is the preservation of its trypsin-interaction site at the N-terminal part of the molecule, with Lys17 at the P1 position. Many trypsin-inhibiting KSPIs interact with coagulation factors, for example thrombin, FXa and FXIa, and therefore express antithrombotic and anticoagulant activities [43]. 

We observed extensive differences in venom proteomes between *V. ursinii* ssp. snakes living in the wild and those living in captivity (Figure 5; Table 2 and Table S2). This finding was, however, expected given that the animals in captivity had exclusively been fed on mice, as opposed to the primarily insectivorous diet of wild *V. ursinii* ssp. A shift in the venom composition due to a diet change has been described before [45]. In our case, the most prominent difference between the *Vu*Cro and *Vu*Cro-c venom proteomes lay in the content of sPLA_2_s. This finding supports the hypothesis that the acquisition of diverse venom sPLA_2_ isozymes by accelerated evolution [46] represents a strong selective advantage for diverse physiological functions, including rapid adaptation to available prey through changes in gene expression [47].

## 4. Conclusions

*V. ursinii* ssp. is, ecologically, a very special viper. Its reliance on insect rather than rodent prey is also reflected in the distinctively higher toxicity of its venom for insects than for mice as compared with venom of a closely related, typical mouse-feeding snake, *V. a. ammodytes*. *V. ursinii* ssp. venom is, however, as haemorrhagic to mice as venom from *V. a. ammodytes*. The *V. ursinii* ssp. venom proteome is also much less complex than that of *V. a. ammodytes*, containing representatives of only seven different protein families compared to 16 in *V. a. ammodytes*. In addition, most of these families contain fewer protein isoforms in *V. ursinii* ssp. compared to *V. a. ammodytes*. SVMPs, which are the most abundant proteins in the *V. ursinii* ssp. venom, are most likely responsible for its haemorrhagic property. The insecticidal activity also suggests the existence of insect-specific toxins in the *V. ursinii* ssp. venom, which may have potential as bioinsecticides. 

## 5. Materials and Methods 

### 5.1. Reagents and Chemicals

Bovine serum albumin (BSA), Tween 20 and *o*-phenylenediamine dihydrochloride (OPD) were from Sigma-Aldrich, Saint Louis, MO, USA. Horseradish peroxidase-conjugated goat anti-rabbit IgG (HRP-anti-rabbit IgG) was from Bio-Rad Laboratories, USA. Iodoacetamide (IAA), 1,4-dithio-DL-threitol (DTT), α-cyano-4-hydroxycinnamic acid (CHCA), Coomassie Brilliant Blue R250 (CBB R250), and all peptide standards were from Sigma, USA. Chemicals for buffers and solutions were from Kemika, Zagreb, Croatia, unless otherwise stated. Water for injection (WFI) was from the Croatian Institute of Transfusion Medicine, Zagreb, Croatia.

### 5.2. Snake Venoms and Antivenoms

A pooled sample of *V. ursinii* ssp. venom was obtained by milking more than 10 adult snakes caught at an isolated locality of the southern Velebit, identified by an expert herpetologist and then released. A small amount of venom was also collected from 4 adult *V. ursinii* ssp. snakes kept in captivity for research purposes at Zagreb Zoo Research and Conservation Centre, and designated as *Vu*Cro-c. Two of these (male and female) were captured 6 years ago, and, since then, fed exclusively on mice. Two were however born in captivity and have never been fed on insects. Crude venom of *V. a. ammodytes* was collected by milking snakes kept at the Institute of Immunology Inc., Zagreb, Croatia. All *V. ursinii* ssp. and *V. a. ammodytes* venom samples were air dried at room temperature and stored in the dark at +4 °C until use. 

Rabbit serum against the whole *V. a. ammodytes* venom (anti-*Vaa*) and against *V. a. ammodytes* venom’s components: haemorrhagins (anti-H), Atx (anti-Atx) and AtnI_2_ (anti-AtnI_2_) were produced on a small scale according to the in house immunisation scheme [48,49].

### 5.3. Animals for In Vivo Assays

All animal procedures were approved by the Croatian Ministry of Agriculture, Veterinary and Food Safety Directorate (UP/I-322-01/17-01/75, permission no. 525-10/0255-17-6, date 12 December 2017), the Animal Protection Department and the University of Zagreb’s Animal Welfare Committee. Animal work was in accordance to Croatian Low on Animal Welfare (2017), which complies strictly with the EC Directive (2010/63/EU) and the ARRIVE guidelines for the Report for in vivo experiments [50]. The approval is based on the positive opinion of the National Ethical Committee (EP 110/2017, date 14 September 2017).

Mice and rats used for in vivo assays were bred at the Institute of Immunology Inc., Croatia. Mice used for the assay of lethal toxicity were of the NIH Ola/Hsd strain. Rats of the Lewis strain were used to assess haemorrhagic activity. Animals were housed under a 12 h light/dark cycle at a temperature of 23 ± 3 °C. A standard mouse/rat diet (Mucedola srl, Milano, Italy) and water were supplied ad libitum during the whole period of experiments.

### 5.4. Assay of Lethal Toxicity in Mice

Lethal toxicity, expressed as the median lethal dose (LD_50_), was determined in adult male NIH Ola/Hsd mice according to the method of Theakston and Reid [51] and European Pharmacopeia (Ph.Eur.01/2008:0145). Groups of six animals (18–20 g each) were injected i.v. with 250 µL of the respective venom solution in saline. Venom doses ranged from 5.25 to 25 µg per mouse. Mortality was recorded after 48 h. For each venom sample, the assay was repeated at least twice and up to five times. The median lethal dose was calculated by Spearman–Kärber analysis. The results were expressed as the mean of *n* determinations ± standard error (SE).

### 5.5. Assay of Lethal Toxicity in Crickets

The entomotoxic effects of *V. ursinii* ssp. and *V. a. ammodytes* venoms were investigated on crickets (*Gryllus assimilis*) as described [7]. Crickets (0.50–0.95 g each), in groups of 4, were injected with 5 µL of solutions in water for injection (WFI) of the respective venom. Venom doses ranged from 1.56 to 100 µg per cricket. An equal volume of WFI was injected into a control group. Mortality was recorded after 48 h. The median lethal dose was calculated by Spearman-Kärber analysis. The results were given as the mean of *n* determinations ± SEM.

### 5.6. Assay for Assessing Haemorrhagic Activity

Haemorrhagic activity was estimated according to Theakston and Reid [51], with experimental details as described in [52]. Briefly, Lewis rats (250 g) were shaved on the dorsal skin and injected i.d. with 100 μL of the venom solution in saline. Six doses, spanning from 1.28 to 50 µg, were tested on each animal. The haemorrhagic lesions on the inner surface of the removed skin were observed 24 h later and their perpendicular diameters measured, from which the average value for each venom dose was calculated. The minimal haemorrhagic dose (MHD) of the venom is defined as the amount of venom (µg dry mass) which, when injected intradermally, 24 h later results in a haemorrhagic lesion of 10 mm in diameter. Results are given as means of *n* determinations ± SEM.

### 5.7. Immunochemical Assays

#### 5.7.1. ELISA Assay

ELISA assays for detecting haemorrhagin-, Atx- or Atn-like proteins in *V. ursinii* ssp. venom were performed as described [53]. Briefly, microtiter plates were coated with 100 µL/well of the venom solution (1 µg/mL) in 50 mM carbonate buffer, pH 9.6, and left overnight at room temperature (RT). After washing and blocking with 0.5% (m/v) BSA in PBS/T (0.05% (v/v) Tween 20 in PBS) buffer (200 µL/well) at 37 °C for 2 h. The rabbit antisera against whole *V. a. ammodytes* venom (anti-*Vaa*) or its components (anti-H, anti-Atx or anti-AtnI_2_) were added in 2-fold serial dilutions (100 µL/well) in duplicate and left overnight at RT. In the subsequent steps, incubation with HRP-anti-rabbit IgG (100 µL/well of a 10,000-fold dilution) at 37 °C for 2 h took place, followed by the addition of 100 µL/well of OPD solution (0.6 mg/mL with 0.015% H_2_O_2_ (v/v) in citrate–phosphate buffer, pH 5.0). After 30 min incubation in the dark, the enzymatic reaction was stopped with 1 M H_2_SO_4_ (50 µL/well) and the absorbance at 492 nm measured.

#### 5.7.2. One-Dimensional SDS-PAGE (1-DE) and Western Blot

1-DE analysis of *V. ursinii* ssp. and *V. a. ammodytes* venoms was performed on 4–12% Bis-Tris precast gels with MES as running buffer, under both reducing and non-reducing conditions at 180 V for 50 min in an Xcell SureLock Mini-Cell, according to the manufacturer’s instructions (Invitrogen, Carlsbad, CA, USA). A total of 40 µg of respective venom was applied in each well. Molecular mass standards were from Invitrogen. Protein staining was carried out with CBB R250. For Western blots (WB) following SDS-PAGE analysis, venoms were electro-blotted to the PVDF membrane (GE Healthcare, Buckinghamshire, UK) in an Xcell Sure Lock Mini Cell according to the manufacturer’s procedure (Invitrogen, Carlsbad, CA, USA). The blocking was performed with 2% (m/v) BSA in PBS/T buffer at +4 °C overnight. The blotted membrane was incubated first with anti-Atx serum (diluted 20,000-fold) and then with HRP-anti-rabbit IgG (diluted 10,000-fold) at 37 °C for 1 h. The Enhanced ChemiLuminescence (ECL) plus Western Blotting Detection System was used for detection, according to the manufacturer’s instructions (GE Healthcare, Buckinghamshire, UK).

### 5.8. Two-Dimensional Gel Electrophoresis (2-DE)

In the first dimension, isoelectric focusing of *V. a. ammodytes* and *V. ursinii* ssp. venoms was performed in a ZOOM IPG Runner Mini-Cell, using immobilised pH gradient (IPG) strips (7 cm long, non-linear pH 3–10) previously rehydrated with the protein sample (250 μg), according to the manufacturer’s procedure (Invitrogen, Carlsbad, CA, USA). The following voltage protocol was applied: 200 V for 20 min, 450 V for 15 min, 750 V for 15 min and 2000 V for 5 h. The focused IPG strips were first reduced with 20 mM DTT, then alkylated with 125 mM IAA. Cys residues in proteins are namely not stable, which hinders their precise analysis. Reaction of Cys residues with IAA derivatizes them to S-carbamidomethyl-Cys residues, which are, however, stable. Each step was performed for 15 min at RT. In the second dimension, SDS-PAGE was performed as described under 5.7.2. For spot detection and quantification, the gel image obtained by an Image Scanner using LabScan 5 software was analysed by Image Master 2D Platinum 6.0 software (GE Healthcare, Amersham Biosciences, Amersham, UK). The 2-DE spot intensity was calculated by integrating the optical density over the spot area.

### 5.9. Mass Spectrometry (MS) Analysis

*V. ursinii* ssp. venom proteins, separated by 1-DE or 2-DE, were excised from the gel and digested with trypsin. The resulting peptides were extracted from the gel and analysed on an ESI–IT mass spectrometer, 1200 series HPLC-Chip-LC/MSD Trap XCT Ultra (Agilent Technologies, Waldbronn, Germany), as described in [54]. Prior to MS analysis, peptides were purified on self-prepared Stage-Tips. Proteins were identified using licensed software MASCOT version 2.1 (Matrix Science, London, UK), searching the “Snakes” (taxid 8750; 159,187 entries) protein database extracted from the non-redundant NCBI (National Centre for Biotechnology Information) protein databank in December 2017 and supplemented with the *V. a. ammodytes* venom gland cDNA transcripts library [55]. The following search parameters were used: parent ion error tolerance of 1.2 Da, fragment error tolerance of 0.60 Da, maximum of 2 missed cleavages allowed, peptide charges +2 and +3, methionine oxidation as variable and carboxamidomethyl cysteine as fixed modification, and an automatic decoy database search. The results were validated using Scaffold software (version 2, Proteome Software, Inc., Portland, OR, USA) with the following parameters: protein threshold of 95%, minimum one peptide per protein and a peptide threshold of 90%. The estimated Prophet false discovery rate was 0.2% for proteins and 5.6% for peptides. Additional peptide matches to identified proteins were obtained by performing an error-tolerant Mascot search and manual validation of the obtained results.

## Figures and Tables

**Table 1:** Lethal toxicity and haemorrhagic potency of the Croatian *V. ursinii* ssp. (*Vu*Cro) and *V. a. ammodytes* (*Vaa*) venoms. Results are given as the mean of *n* determinations ± SEM. MHD stands for minimal haemorrhagic dose.

	*Vu*Cro Venom	*Vaa* Venom
**Lethal Toxicity**		
LD_50_ in mice (µg)	37.0 ± 0.1 (*n* = 2)	4.4–13.7 *
** Mass-normalized LD_50_ in mice (µg/g)	1.94	0.47
LD_50_ in crickets (µg)	7.1 ± 0.4 (*n* = 2)	38.7 ± 3.3 (*n* = 2)
** Mass-normalized LD_50_ in crickets (µg/g)	9.8	53.4
**Haemorrhagic Activity**		
MHD in rats (µg)	34.1 ± 4.8 (*n* = 4)	21.6–42.8*

* Depending on geographical location (determined by Halassy et al. [13,14]). ** Mass-normalized LD_50_ values were calculated as the average LD_50_ (in µg)/average body mass of experimental animal (in g) (0.725 g for crickets; 19 g for mice).

**Table 2:** Proteins with a higher or lower abundance in the venom of the Croatian *V. ursinii* ssp. snakes living in captivity (*Vu*Cro*-*c) than in the venom of the snakes living in the wild (*Vu*Cro). Abbreviations: AtnI, ammodytin I; CRISP, cysteine-rich secretory protein; KSPI, Kunitz-type serine protease inhibitor; sPLA_2_, secreted phospholipase A_2_; SVMP, snake venom metalloproteinase; SVSP, snake venom serine protease; VNGF, venom nerve growth factor; *Vaa*, *V. a ammodytes*; *Vu*, *V. ursinii*.

Higher Abundance	Protein Family
AtnI_1_ (B) isoform (*Vu*)	sPLA_2_
Vur-PL2	sPLA_2_
AtnI2 (D) isoform (*Vu*)	sPLA_2_
RVV-X light chain 2 (*Daboia siamensis*)	snaclec
Vaa-SP-3 *	SVSP
Vaa-SP-4	SVSP
Vaa-SPH-1 (*Vaa*)	SVSP
VNGF (*Vu*)	VNGF
Vur-KIn (*V. renardi*)	KSPI
**Lower Abundance**	
Metalloproteinases	SVMP
Vaa-CRISP-1 (*Vaa*)	CRISP

* Not identified in the *Vu*Cro venom.

## Supplementary Materials

The following are available online at https://www.mdpi.com/2072-6651/12/3/187/s1, Table S1: Identification of the structures of the Croatian *V. ursinii* ssp. venom proteins. Table S2: Identification of proteins with higher abundance in the venom of the Croatian *V. ursinii* ssp. snakes living in captivity (VuCro-c) than in the venom of those living in the wild (VuCro).
