# Supplementary material for: Biological Activities and Proteomic Profile of the Venom of Vipera ursinii ssp., a very Rare Karst Viper from Croatia
Source: Toxins (Basel). 2020 Mar 16;12(3):187. doi: 10.3390/toxins12030187 (PMC7150868; doi:10.3390/toxins12030187)
Supplement: Supplementary file 1 [file toxins-12-00187-s001.pdf]

Supplementary Materials: Biological Activities and Proteomic Profile of the Venom of *Vipera ursinii* ssp., a very Rare Karst Viper from Croatia

Maja Lang Balija, Adrijana Leonardi, Marija Brgles, Dora Sviben, Tihana Kurtović, Beata Halassy * and Igor Križaj *

Table S1. Identification of the structures of the Croatian *V. ursinii* ssp. venom proteins. The venom was separated by gel electrophoresis in one (1-DE) or in two (2-DE) dimensions. Cys in proteins, separated in bands and spots (labelled as in Figures 4A and 4B), were first carbamidomethylated and then digested in-gel by trypsin. The resulting peptides were analysed on LC-ESI-MS/MS. Modified (apart from Cys) or mutated amino acid residues are typed in bold letters. Abbreviations: AtnI, ammodytin I; CRISP, cysteine-rich secretory protein; FIXa, activated factor IX; FX, factor X; KSPI, Kunitz-type serine protease inhibitor; n.d., not determined; sPLA$_2$, secreted phospholipase A$_2$; SVMP, snake venom metalloproteinase; SVSP, snake venom serine protease; *Vaa*, *V. a. ammodytes*; VNGF, venom nerve growth factor; *Vu*, *V. ursinii*; *, by similarity. ->, substitution of amino acid residue on the left with that on the right.

1-DE-Band/ 2-DE-Spot	Mol. Mass (kDa)	MS/MS-Derived Peptide Sequence	Predicted Sequence Modification	Peptide Mass (Da)	Protein Identity (Snake Species)	Protein ID	Protein Score	Protein Activity (Reference)	Protein Family
1DE-1	80	IPCAPQDVK		1041.54	VaH3 (*Vaa*)	AGL45259	178	haemorrhagin [1]	SVMP
		LGNEYGYCR		1131.49					
		SCIMSGTLSCE**A**SIR	Ala->Gly	1673.73					
		ATVAEDSCFQENQK		1626.71	VLFXA heavy chain (*M. lebetina*)	AAQ17467	129.5	FX activator [2]	SVMP
		KIPCAPQDVK		1169.64					
		CILNPPLR		982.55					
		KIPCAPQDIK		1155.62	RVV-X heavy chain (*D. russelii*)	Q7LZ61	34.0	FX activator [3]	SVMP
1DE-2	75	DCQNPCCNAATCK		1598.58	VaH3 (*Vaa*)	AGL45259	528	haemorrhagin [1]	SVMP
		KIPCAPQDVK		1155.62					
		LGNEYGYCR		1131.49					
		LTPGSQCADGECCDQCK		1985.75					
		**M**PQCILNKPLK	Met oxidation	1357.73					

		NPCQIYYTPR		1369.62					
		SC**I**MSGTLSCEASIR	Ile->Val	1657.73					
		YLVELGEDCDCGSPR		1769.75					
		ATVAEDSCFQENQK		1626.71	VLFXA heavy chain (*M. lebetina*)	AQ17467	172	FX activator [2]	SVMP
		DCQNPCC**D**AATCK	Asp->Asn	1598.58					
		KIPCAPQDIK		1155.62	RVV-X heavy chain (*D. russelii*)	Q7LZ61	48.8	FX activator [3]	SVMP
1DE-3	48	IYEMVNTLNVVFR		1597.84	Vaa-MPIII-1 (*Vaa*)	AMB36352	321.0	n.d.	SVMP
		LVATSEQQSYYDR		1559.73					
		LVIVVDHSMVTK		1340.76					
		VATSEQQSYYDR		1446.65					
		YNNNST**A**IR	Ala->Val	1080.54					
		LHSWVECESGECCEQCR		2283.85	HR1a (*P. mucrosquamatus*)	Q8JIR2	166.9	haemorrhagin [4]	SVMP
		SWVECESGECCEQCR		1975.70					
		GEECDCGSPANCQDPCC DA**A**SCK	Ala->Thr	2677.90	SVMP (*E. c. sochureki*)	ADI47594	150.8	n.d.	SVMP
		KGKSYFYCR		1208.59					
		SECDLPEYCTGQ		1458.55					
		LVIVVDHSMVEK		1368.76	carinactivase-1 (*E. carinatus*)	Q9PRP9	46.3	prothrombin activator [5]	SVMP
1DE-4	45	LVATSEQQSYYDR		1559.73	Vaa-MPIII-1 (*Vaa*)	AMB36352	306.1	n.d.	SVMP
		SQLVATSEQQSYYDR		1774.82					
		VATSEQQSYYDR		1446.65					
		IYEMVNTLNVVFR		1597.84					
		FLTNFKPDCTLIR		1624.85	SVMP (*E. c. sochureki*)	ADI47595	132.5	n.d.	SVMP
		FLTNFKPDCTLIRPSR		1965.99					
		TDIVSPPVCGNDL**L**ER	Leu->Val	1770.87					
		LHSWVECESGECCEQCR		2283.85	HR1a (*P. mucrosquamatus*)	Q8JIR2	124.0	haemorrhagin [4]	SVMP
		SWVECESGECCEQCR		1975.70					
		LVIVVDHSMVEK		1367.89	carinactivase-1 (*E. carinatus*)	Q9PRP9	45.4	prothrombin activator [5]	SVMP

		MEWYPEAAANAER		1537.67	Vaa-CRISP-1 (*Vaa*)	KT148819	153.6	n.d.	CRISP
		VIGGIECGENIYMSTSPMK		2086.93					
		VIGGDECNINEHR		1512.69	Vaa-SP-4 (*Vaa*)	KT148827	49.5	n.d.	SVSP
1DE-5	35	**E**KFFCLSSK	Glu->Gln	1144.58	Vaa-SPH-1 (*Vaa*)	KT148824	310.6	FIXa antagonist [6]	SVSP
		IMGWGTITTTK		1208.64					
		NVPNEDEQ**M**R	Met oxidation	1247.53					
		NVPNEDEQMRVPK		1556.74					
		SLPSSPPSVGSVCR		1429.71					
		TLCAGILQGGIDSCK		1675.85					
		QKFFCLSSK		1145.57	Vaa-SP-6 (*Vaa*)	MG958495	97.5	FV, FX activator [Latinović et al., in preparation]	SVSP
		VVCAGIWQGGK		1174.60					
		NIRNEDEQIR		1286.65	RVV-V alpha (*D. siamensis*)	P18964	85.7	FV activator [7]	SVSP
		YFCLNTK		929.45					
		SVDFDSESPR		1138.50	Vaa-CRISP-1 (*Vaa*)	KT148819	50.6	n.d.	CRISP
1DE-6	27	C**T**YDHSPR	Thr->Ile	1047.47	Vaa-CRISP-1 (*Vaa*)	KT148819	635.0	n.d.	CRISP
		DFVYGQGASPANAVVGH		1688.80					
		DFVYGQGASPANAVVGHYT		1952.91					
		KPEIQNEIIDLHNSLR		1920.02					
		**M**EWYPEAAANAER	Met oxidation	1553.67					
		RSVNPTASNMLK		1316.71					
		SVDFDSESPR		1138.50					
		SVNPTASNMLK		1161.59					
		VIGGIECGENIYM		1454.67					
		VIGGIECGENIYMSTSPMK		2085.97					
		YTQIVWYK		1100.58					
		DFVYGQGASPANAVVGH		1688.80	Dr-CRPK (*D. russelii*)	ACE73567	445.6	n.d.	CRISP

		DFVYGQGASPANAVVGHYT		1952.91					
		KDFVYGQGASPANAVVGH		1816.90					
		KDFVYGQGASPANAVVGHYT		2081.01					
		MEWYPEAAANAER		1553.67					
		RSVTPTASNMLK		1333.73					
		SVDFDSESPR		1138.50					
		YTQIVWYK		1100.58					
		FYCAGTLINQEWVLTAAR		2113.05	Vaa-SPH-1 (*Vaa*)	KT148824	173.6	FIXa antagonist [6]	SVSP
		IMGWGTITTTK		1208.64					
		TLCAGILQGGIDSCK		1592.78					
1DE-7	14	CCFVHDCCYGR		1533.55	AtnI$_1$ (B) isoform (*Vu*)	CAE47156	298.0	indirect haemolytic activity * [8]	sPLA$_2$
		NLSQFGDMINK		1266.62					
		VAAICFGENMNTYDK		1732.77					
		VAAICFGENMNTYDKK		1861.85					
		YMLYSLFDCK		1339.61					
		IYE**M**VNTLNVVFR	Met oxidation	1613.84	Vaa-MPIII-1 (*Vaa*)	KT148834	128.0	n.d.	SVMP
		LVIVVDHSMVTK		1340.76					
1DE-8	60	IYEMVNTLNVVFR		1597.84	Vaa-MPIII-1 (*Vaa*)	KT148834	306.7	n.d.	SVMP
		LVATSEQQSYYDR		1559.73					
		LVIVVDHSMVTK		1340.76					
		VATSEQQSYYDR		1446.65					
		YNNNST**A**IR	Ala->Val	1080.54					
		LVATSEQQSYYDR		1559.73	Vaa-MPIII-4 (*Vaa*)	MG958500	240	n.d.	SVMP
		VATSEQQSYYDR		1446.65					
		VNILNEMYLPLNIR		1701.94					
		YIKLVIVVDHR		1355.77					

		YDYSEDPDYGMVDHGTK		1990.81	Vaa-MPIII-3 (*Vaa*)	MG958499	47.2	n.d.	SVMP
1DE-9	18	ALTMEGNQASWR		1379.64	VNGF (*Vu*)	AEH59582	130	promotes nerve growth * [9]	VNGF
		IDTACVCVISR		1293.63					
		LVIVVDHSMVTK		1340.76	Vaa-MPIII-1 (*Vaa*)	KT148834	118.8	n.d.	SVMP
		VATSEQQSYYDR		1446.65					
		NYPSSECTETEQC		1604.58	$AtnI_2$ (D) isoform (*Vu*)	CAE47222	106.4	anticoagulant, antiplatelet* [10]	$sPLA_2$
		VAAICFGENLNTYDK		1714.81					
1DE-10	13	FIYGGCR		872.41	chymotrypsin inhibitor (*Vaa*)	0909196A	78.0	protease inhibitor [11]	KSPI
		FYYNPASNK		1103.52					
		EFFYGGCGGNANNFK		1681.71	Vur-KIn (*V. renardi*)	P0DKL8	55.0	protease inhibitor [10]	KSPI
2DE-1	53	IYEMVNTLNVVFR		1597.84	Vaa-MPIII-1 (*Vaa*)	KT148834	112.7	n.d.	SVMP
		LVIVVDHSMVTK		1340.76					
		KENDVPIPCAPEDIK		1724.85	Vaa-MPIII-3 (*Vaa*)	MG958499	69.52	n.d.	SVMP
		YDYSEDPDYG**M**VDHGTK	Met oxidation	2007.79					
		YIKLVIVVDHR		1355.77	Vaa-MPIII-4 (*Vaa*)	MG958500	64.7	n.d.	SVMP
2DE-2	53	ATSEQQSYYDR		1347.58	Vaa-MPIII-1 (*Vaa*)	KT148834	355.4	n.d.	SVMP
		IYE**M**VNTLNVVFR	Met oxidation	1613.84					
		LVATSEQQSYYDR		1559.73					
		LVIVVDHSMVTK		1340.76					
		TDIVSPPVCGNELLEK		1770.90					
		VATSEQQSYYDR		1446.65					
		KIPCAPQDVK		1155.62	VaH3 (*Vaa*)	AGL45259	96.8	haemorrhagin [1]	SVMP
		NPCQIYYTPR		1311.62					
		LVIVVDHS**M**VLK	Met oxidation	1368.79	carinactivase-1 (*E. carinatus*)	AAB36410	42	prothrombin activator [5]	SVMP

2DE-3	120	YDYSEDPDYG**M**VDHGTK	Met oxidation	2007.79	Vaa-MPIII-3 (*Vaa*)	MG958499	125.6	n.d.	SVMP
		KENDVPIPC**A**PEDIK	Ala->Ser	1740.85					
		LHSWVECESGECCEQCR		2283.85	HR1a (*P. mucrosquamatus*)	Q8JIR2	64.5	haemorrhagin [4]	SVMP
2DE-5	120	IYE**M**VNTLNVVFR	Met oxidation	1613.84	Vaa-MPIII-1 (*Vaa*)	KT148834	243.0	n.d.	SVMP
		LVIVVDHS**M**VTK	Met oxidation	1340.76					
		SQLVATSEQQSYYDR		1774.82					
		TDIVSPPVCGNELLEK		1770.90					
		FRYIKFVIVVDHSMVEK		2091.1	SVMP (*E. p. leakeyi*)	ADI47674	163	n.d.	SVMP
		FVIVVDHSMVEK		1368.76					
		SQLVATSEQQSYYDR		1774.82					
		KENDVPIPCAPEDIK		1724.85	Vaa-MPIII-3 (*Vaa*)	MG958499	73.6	n.d.	SVMP
		YDYSEDPDYGMVDHGTK		1991.80					
		LVIVVDHS**M**VEK	Met oxidation	1384.75	carinactivase-1 (*E. carinatus*)	AAB36410	43.8	prothrombin activator [5]	SVMP
		IMGWGTITTTK		1224.63	Vaa-SPH-1 (*Vaa*)	KT148824	103.2	FIXa antagonist [6]	SVSP
		TLCAGILQGGIDSCK		1592.78					
2DE-7	120	IYEMVNTLNVVFR		1613.84	Vaa-MPIII-1 (*Vaa*)	KT148834	51.6	n.d.	SVMP
2DE-10	120	IYEMVNTLNVVFR		1613.84	Vaa-MPIII-1 (*Vaa*)	KT148834	166.3	n.d.	SVMP
		LVIVVDHSMVTK		1340.76					
		TDIVSPPVCGNELLEK		1770.90					
		KENDVPIPCAPEDIK		1724.85	Vaa-MPIII-3 (*Vaa*)	MG958499	135.3	n.d.	SVMP
		YDYSEDPDYGMVDHGTK		1990.81					
		LVIVVDHSMVTK		1340.76					
		FLTDFKPDCTLIRPSR		1966.02	Vaa-MPIII-4 (*Vaa*)	MG958500	91.8	n.d.	SVMP
		VNILNEMYLPLNIR		1701.94					
		LHSWVECESGECCEQCR		2283.85	HR1a (*P. mucrosquamatus*)	Q8JIR2	74.4	haemorrhagin [4]	SVMP
		IMGWGTITTTK		1208.64	Vaa-SPH-1 (*Vaa*)	KT148824	101.2	FIXa antagonist [6]	SVSP
		TLCAGILQGGIDSCK		1593.74					
		KPEIQNEIIDLHNSLR		1920.02	Vaa-CRISP-1 (*Vaa*)	KT148819	84.3	n.d.	CRISP

		**M**EWYPEAAANAER	Met oxidation	1553.67					
2DE-16	54	DFVYGQGASPANAVVGH		1688.80	Vaa-CRISP-1 (*Vaa*)	KT148819	255.4	n.d.	CRISP
		DFVYGQGASPANAVVGH YTQIVWYK		2770.36					
		KPEIQNEIIDLHNSLR		1920.02					
		MEWYPEAAANAER		1562.74					
		VIGGIECGENIYMSTSPMK		2085.97					
		IYE**M**VNTLNVVFR	Met oxidation	1613.84	Vaa-MPIII-1 (*Vaa*)	KT148834	101.7	n.d.	SVMP
		TDIVSPPVCGNELLEK		1770.90					
		TLCAGILQGGIDSCK		1592.78	Vaa-SPH-1 (*Vaa*)	KT148824	81.4	FIXa antagonist [6]	SVSP
		VTYPDVPHCADINMFDYS VCQK		2659.6					
2DE-21	48	AENPWLPAQSR		1294.65	Vaa-SP-4 (*Vaa*)	KT148827	291.6	n.d.	SVSP
		IAPLSLPSSPPR		1234.72					
		INILNYAVCR		1235.66					
		STHIAPLSLPSSPPR		1559.85					
		SYTLWNKDIMLIK		1625.86					
		TLCAGILQGGIDTCK		1606.79					
		SYTLWDKDI**M**LIK	Met oxidation	1641.21					
		LHSWVECESGECCEQCR		2226.83	HR1a (*P. mucrosquamatus*)	Q8JIR2	53.7	haemorrhagin [4]	SVMP
2DE-22	38	AENPWLPAQSR		1310.65	Vaa-SP-4 (*Vaa*)	KT148827	125.9	n.d.	SVSP
		TLCAGILQGGIDTCK		1606.79					
		TLCAGILQGGIDSCK		1592.78	Vaa-SPH-1 (*Vaa*)	KT148824	82.4	FIXa antagonist [6]	SVSP
2DE-23	50	FRYIKLVIVVDHSMVTK		2050.8	Vaa-MPIII-1 (*Vaa*)	KT148834	133.8	n.d.	SVMP
		LVIVVDHSMVTK		1340.76					
		TDIVSPPVCGNELLEK		1770.90					
2DE-24	48	IYEMVNTLNVVFR		1613.84	Vaa-MPIII-1 (*Vaa*)	KT148834	116.4	n.d.	SVMP
		LVIVVDHSMVTK		1340.76					
		KENDVPIPCAPEDIK		1724.85	Vaa-MPIII-3 (*Vaa*)	MG958499	94.3	n.d.	SVMP

		LVIVVDHS**M**VTK	Met oxidation	1340.76					
2DE-25	48	IYE**M**VNTLNVVFR	Met oxidation	1613.84	Vaa-MPIII-1 (*Vaa*)	KT148834	160.7	n.d.	SVMP
		LVIVVDHSMVTK		1340.76					
		TDIVSPPVCGNELLEK		1770.90					
		LHSWVECESGECC**QQ**CR	deamidation	2226.83	SVMP (*E. coloratus*)	ADI47644	112.7	n.d.	SVMP
		LVIVVDHSMVTK		1340.76					
		LVIVVDHSMVEK		1368.76	carinactivase-1 (*E. carinatus*)	Q9PRP9	45.5	prothrombin activator [5]	SVMP
		KE**N**DVPIPCAPEDIK	deamidation	1725.81	Vaa-MPIII-3 (*Vaa*)	MG958499	35.9	n.d.	SVMP
2DE-26	37	IMGWGTITTTK		1208.64	Vaa-SPH-1 (*Vaa*)	KT148824	154.4	FIXa antagonist [6]	SVSP
		TLCAGILQGGIDSCK		1592.78					
		VTYPDVPHCADINMFDYSVCQK		2659.6					
		IYE**M**VNTLNVVFR	Met oxidation	1613.84	Vaa-MPIII-1 (*Vaa*)	KT148834	101.2	n.d.	SVMP
		TDIVSPPVCGNELLEK		1770.90					
		VAAICFGENLNTYDK		1714.81	AtnI$_2$ (D) isoform (*Vu*)	CAE47222	43.7	anticoagulant, antiplatelet* [10]	sPLA$_2$
2DE-30	35	SLPSSPPSVGSVCR		1429.71	Vaa-SPH-1 (*Vaa*)	KT148824	101.8	FIXa antagonist [6]	SVSP
		TLCAGILQGGIDSCK		1592.78					
		SEWSDGSSVSYDNL**L**K	Leu->His	1810.79	RVV-X light chain 2 (*D. siamensis*)	ADK22819	55.5	FX activator [3]	snaclec
2DE-31	30	LTIYSYSFENGDIVCGGDDSCKR		2656.7	Vur-PL2 (*V. renardi*)	ADG86231	147.4	anticoagulant, anti-platelet [10]	sPLA$_2$
		VAAICFGENLNTYDK		1714.81					
		KENDVPIPCAPEDIK		1724.85	Vaa-MPIII-3 (*Vaa*)	MG958499	95.6	n.d.	SVMP
		YDYSEDPDYG**M**VDHGTK	Met oxidation	2007.79					

		TLCAGILQGGIDSCK		1592.78	Vaa-SPH-1 (*Vaa*)	KT148824	45	FIXa antagonist [6]	SVSP
2DE-32	35	I**M**GWGTITTTK	Met oxidation	1224.63	Vaa-SPH-1 (*Vaa*)	KT148824	99.9	FIXa antagonist [6]	SVSP
		TLCAGILQGGIDSCK		1592.78					
		LVIVVDHSMVTK		1356.76	Vaa-MPIII-1 (*Vaa*)	KT148834	70.2	n.d.	SVMP
		YNNNST**A**IR	Ala->Val	1080.54					
2DE-33	30	VAAICFGENLNTYDK		1714.81	AtnI$_2$ (D) isoform (*Vu*)	CAE47222	59	anticoagulant, antiplatelet* [10]	sPLA$_2$
		TLCAGILQGGIDSCK		1592.78	Vaa-SPH-1 (*Vaa*)	KT148824	48.3	FIXa antagonist [6]	SVSP
		MEWYPEAAANAER		1553.67	Vaa-CRISP-1 (*Vaa*)	KT148819	46	n.d.	CRISP
2DE-38	20	VAAICFGENLNTYDK		1714.81	AtnI$_2$ (D) isoform (*Vu*)	CAE47222	122	anticoagulant, antiplatelet* [10]	sPLA$_2$
		VAAICFGENLNTYDKK		1842.91					
		SEWSDGSSVSYDNL**L**K	Leu->His	1809.84	RVV-X light chain 2 (*D. siamensis*)	ADK22819	62.2	FX activator [3]	snaclec
2DE-41	14	LTIYSYSFENGDIVCGGDDSCKR		2657.5	Vur-PL2 (*V. renardi*)	ADG86231	196.0	anticoagulant, anti-platelet [10]	sPLA$_2$
		VAAICFGENLNTYDK		1714.41					
		VAAICFGENLNTYDKK		1900.91					
2DE-43	14	LTIYSYSFENGDIVCGGDDSCKR		2656.7	Vur-PL2 (*V. renardi*)	ADG86231	225.0	anticoagulant, anti-platelet [10]	sPLA$_2$
		VAAICFGENLNTYDK		1714.81					
		VAAICFGENLNTYDKK		1842.91					
		VEAICFGENLNTYDK		1715.80	AtnI$_2$ (A) isoform (*V. a. ruffoi*)	CAE47218	177	anticoagulant, antiplatelet* [10]	sPLA$_2$
		VEAICFGENLNTYDKK		1842.94					
		VAAICFGEN**M**NTYDK	Met oxidation	1748.76	AtnI$_1$ (B) isoform (*Vu*)	CAE47156	89.7	indirect haemolytic activity* [8]	sPLA$_2$
		VAAICFGEN**M**NTYDKK	Met oxidation	1876.86					

2DE-44	12	IYYGCYCGWGGK		1483.61	AtnI$_1$ (B) isoform (*Vu*)	CAE47156	275.7	indirect haemolytic activity* [8]	sPLA$_2$
		MGTYSYSFQ**N**GDIVCGGDDPCLR	deamidation	2613.07					
		NLSQFGDMINK		1266.62					
		VAAICFGEN**M**NTYDK	Met oxidation	1748.76					
		VAAICFGENMNTYDKK		1860.86					
		LTIYSYSFENGDIVCGGDDSCKR		2657.5	Vur-PL2 (*V. renardi*)	ADG86231	193.8	anticoagulant, anti-platelet [10]	sPLA$_2$
		VAAICFGENLNTYDK		1714.81					
		VAAICFGENLNTYDKK		1900.91					
2DE-46	14	LTIYSYSFENGDIVCGGDDSCKR		2681.62	Vur-PL2 (*V. renardi*)	ADG86231	227.9	anticoagulant, anti-platelet [10]	sPLA$_2$
		SYSFENGDIVCGGDDSCKR		2165.89					
		VAAICFGENLNTYDK		1714.81					
		VAAICFGENLNTYDKK		1884.92					
		IALFSYSDYGCYCGWGGQGKPK		2514.28	AtnI$_2$ (D) isoform (*Vu*)	CAE47222	182.1	anticoagulant, antiplatelet* [10]	sPLA$_2$
		VAAICFGENLNTYDK		1714.81					
		VAAICFGENLNTYDKK		1884.92					
		KPEIQNEIIDLHNSLR		1919.04	Vaa-CRISP-1 (*Vaa*)	KT148819	100.7	n.d.	CRISP
		**M**EWYPEAAANAER	Met oxidation	1553.67					
2DE-47	12	VAAICFGENLNTYDK		1713.51	AtnI$_2$ (D) isoform (*Vu*)	CAE47222	73.5	anticoagulant, antiplatelet * [10]	sPLA$_2$

Table S2. Identification of proteins with higher abundance in the venom of the Croatian *V. ursinii* ssp. snakes living in captivity (*Vu*Cro-c) than in the venom of those living in the wild (*Vu*Cro). Using comparative 2-DE (Figure 5), the dominant spots of the *Vu*Cro-c venom, as compared to those of the *Vu*Cro venom, were excised (labelled as in Figure 5A). Cys of proteins in spots were carbamidomethylated and digested in-gel by trypsin. The resulting peptides were extracted and analysed by LC-ESI-MS/MS. Apart from Cys, modified or mutated amino acid residues are presented by bold letters. Abbreviations: AtnI, ammodytin I; CRISP, cysteine-rich secretory protein; FX, factor X; n.d., not determined; $sPLA_2$, secreted phospholipase A_2; SVSP, snake venom serine protease; *Vaa*, *V. a. ammodytes*; *Vu*, *V. ursinii*; *, by similarity; ->, substitution of amino acid residue on the left to that on the right.

2-DE-Spot	Protein Mass (kDa)	MS/MS-Derived Sequence	Predicted Sequence Modification	Peptide Mass (Da)	Identified Protein (Snake Species)	Protein ID	Protein Score	Protein Activity (Reference)	Protein Family
1	40	AENPWLPAQSR		1268.64	VaaSP-4 (*Vaa*)	KT148827	167.8	n.d.	SVSP
		SYTLW**N**KDIMLIK	deamidation	1625.86					
		TLCAGILQGGIDTCK		1606.79					
		AA**Y**PWLLER	Tyr->Phe	1102.60	VaaSP-3 (*Vaa*)	KT148826	91.0	n.d.	SVSP
		GQLQGIVSWGYR		1363.71					
2	30	CCFVHDCCYGR		1533.55	$AtnI_2$ (D) isoform (*Vu*)	CAE47222	166.0	anticoagulant, antiplatelet * [10]	$sPLA_2$
		NYPSSECTETEQC		1604.58					
		VAAICFGENLNTYDK		1714.81					
		KPEIQNEIIDLHNSLR		1919.98	VaaCRISP-1 (*Vaa*)	KT148819	108.9	n.d.	CRISP
		RSVNPTASNMLK		1317.69					
		SVNPTASNMLK		1161.59					
		TLCAGILQGGIDTCK		1606.79	VaaSP-4 (*Vaa*)	KT148827	92.7	n.d.	SVSP
		LNRPVTY		862.48					
		KVLNEDEETREPTEK		1816.89	VaaSP-3 (*Vaa*)	KT148826	63.8	n.d.	SVSP
3	18	VAAICFGENLNTYDK		1714.81	$AtnI_2$ (D) isoform (*Vu*)	CAE47222	225.1	anticoagulant, antiplatelet * [10]	$sPLA_2$
		VAAICFGENLNTYDKK		1842.91					
		YKNYPSSECTETEQC		1895.74					
		CCFVHDCCYGR		1533.55					
		SEWSDGSSVSYDNL**L**K	Leu->His	1810.79	RVV-X light chain 2 (*D. siamensis*)	ADK22819	79.4	FX activator [3]	snaclec

4	14	SEWSDGSSVSYDNL**L**K	Leu->His	1810.79	RVV-X light chain 2 (*D. siamensis*)	ADK22819	161.9	FX activator [3]	snaclec
		FITHFWIGLR		1289.72					
		YFCYR		808.34					
		VAAICFGENLNTYDK		1714.81	AtnI$_2$ (D) isoform (*Vu*)	CAE47222	59.7	anticoagulant, antiplatelet * [10]	sPLA$_2$
		NYPSSQCTETEQC		1604.58	Vur-PL2 (*V. renardi*)	ADG86231	44.2	anticoagulant, antiplatelet [10]	sPLA$_2$
5	18	NYPSSECTETEQC		1604.58	AtnI$_2$ (D) isoform (*Vu*)	CAE47222	141.1	anticoagulant, antiplatelet * [10]	sPLA$_2$
		VAAICFGENLNTYDK		1714.81					
		VAAICFGENLNTYDKK		1842.91					
		SEWSDGSSVSYDNL**L**K	Leu->His	1810.79	RVV-X light chain 2 (*D. siamensis*)	ADK22819	104.8	FX activator [3]	snaclec
		YFCYR		808.34					
6	18	CCFVHDCCYGR		1533.55	AtnI$_2$ (D) isoform (*Vu*)	CAE47222	164.7	anticoagulant, antiplatelet * [10]	sPLA$_2$
		VAAICFGENLNTYDK		1714.81					
		VAAICFGENLNTYDKK		1842.91					
		SEWSDGSSVSYDNL**L**K	Leu->His	1810.79	RVV-X light chain 2 (*D. siamensis*)	ADK22819	130.1	FX activator [3]	snaclec
		TWEEAER		920.41					
		YFCYR		808.34					
7	20	CCFVHDCCYGR		1533.55	AtnI$_2$ (D) isoform (*Vu*)	CAE47222	279.7	anticoagulant, antiplatelet * [10]	sPLA$_2$
		IALFSYSDYGCYCGWGGQGKPK		2514.12					
		NYPSSECTETEQC		1604.58					
		VAAICFGENLNTYDK		1714.81					
		VAAICFGENLNTYDKK		1842.91					

References

1. Sajevic, T.; Leonardi, A.; Kovačič, L.; Lang-Balija, M.; Kurtović, T.; Pungerčar, J.; Halassy, B.; Trampuš-Bakija, A.; Križaj, I. VaH3, one of the principal hemorrhagins in *Vipera ammodytes ammodytes* venom, is a homodimeric P-IIIc metalloproteinase. *Biochimie* **2013**, *95*, 1158–1170.
2. Siigur, E.; Tõnismägi, K.; Trummal, K.; Samel, M.; Vija, H.; Subbi, J.; Siigur, J. Factor X activator from *Vipera lebetina* snake venom, molecular characterization and substrate specificity. *Biochim. Biophys. Acta Gen. Subj.* **2001**, *1568*, 90–98.
3. Takeya, H.; Nishida, S.; Miyata, T.; Kawada, S.; Saisaka, Y.; Morita, T.; Iwanaga, S. Coagulation factor X activating enzyme from Russell's viper venom (RVV-X). A novel metalloproteinase with disintegrin (platelet aggregation inhibitor)-like and C-type lectin-like domains. *J. Biol. Chem.* **1992**, *267*, 14109–14117.
4. Kishimoto, M.; Takahashi, T. Molecular cloning of HR1a and HR1b, high molecular hemorrhagic factors, from Trimeresurus flavoviridis venom. *Toxicon* **2002**, *40*, 1369–1375.
5. Yamada, D.; Sekiya, F.; Morita, T. Isolation and characterization of carinactivase, a novel prothrombin activator in *Echis carinatus* venom with a unique catalytic mechanism. *J. Biol. Chem.* **1996**, *271*, 5200–5207.
6. Latinović, Z.; Leonardi, A.; Kovačič, L.; Koh, C.; Šribar, J.; Bakija, A.; Venkateswarlu, D.; Kini, R.; Križaj, I. The first intrinsic tenase complex inhibitor with serine protease structure offers a new perspective in anticoagulant therapy. *Thromb. Haemost.* **2018**, *118*, 1713–1728.
7. Tokunaga, F.; Nagasawa, K.; Tamura, S.; Miyata, T.; Iwanaga, S.; Kisiel, W. The factor V-activating enzyme (RVV-V) from Russell's viper venom. Identification of isoproteins RVV-V alpha, -V beta, and -V gamma and their complete amino acid sequences. *J. Biol. Chem.* **1988**, *263*, 17471–17481.
8. Jan, V.M.; Guillemin, I.; Robbe-Vincent, A.; Choumet, V. Phospholipase A_2 diversity and polymorphism in European viper venoms: Paradoxical molecular evolution in Viperinae. *Toxicon* **2007**, *50*, 1140–1161.
9. Paalme, V.; Trummal, K.; Samel, M.; Tõnismägi, K.; Järvekülg, L.; Vija, H.; Subbi, J.; Siigur, J.; Siigur, E. Nerve growth factor from *Vipera lebetina* venom. *Toxicon* **2009**, *54*, 329–336.
10. Tsai, I.-H.; Wang, Y.-M.; Cheng, A.C.; Starkov, V.; Osipov, A.; Nikitin, I.; Makarova, Y.; Ziganshin, R.; Utkin, Y. cDNA cloning, structural, and functional analyses of venom phospholipases A_2 and a Kunitz-type protease inhibitor from steppe viper *Vipera ursinii renardi*. *Toxicon* **2011**, *57*, 332–341.
11. Ritonja, A.; Turk, V.; Gubenšek, F. Serine proteinase inhibitors from *Vipera ammodytes* venom. Isolation and kinetic studies. *Eur. J. Biochem.* **1983**, *133*, 427–432.
